# Pdlim7 is required for maintenance of the mesenchymal/epidermal Fgf signaling feedback loop during zebrafish pectoral fin development

**DOI:** 10.1186/1471-213X-10-104

**Published:** 2010-10-15

**Authors:** Troy Camarata, Diana Snyder, Tyler Schwend, Julian Klosowiak, Brandon Holtrup, Hans-Georg Simon

**Affiliations:** 1 Department of Pediatrics, Northwestern University, The Feinberg School of Medicine, Children's Memorial Research Center, Chicago, IL (60614), USA; 2 Nephrology Division, Massachusetts General Hospital, Harvard Medical School, Charlestown, MA (02129), USA; 3 Division of Biology, Kansas State University, Manhattan, KS (66502), USA

## Abstract

**Background:**

Vertebrate limb development involves a reciprocal feedback loop between limb mesenchyme and the overlying apical ectodermal ridge (AER). Several gene pathways participate in this feedback loop, including Fgf signaling. In the forelimb lateral plate mesenchyme, Tbx5 activates Fgf10 expression, which in turn initiates and maintains the mesenchyme/AER Fgf signaling loop. Recent findings have revealed that Tbx5 transcriptional activity is regulated by dynamic nucleocytoplasmic shuttling and interaction with Pdlim7, a PDZ-LIM protein family member, along actin filaments. This Tbx5 regulation is critical in heart formation, but the coexpression of both proteins in other developing tissues suggests a broader functional role.

**Results:**

Knock-down of Pdlim7 function leads to decreased pectoral fin cell proliferation resulting in a severely stunted fin phenotype. While early gene induction and patterning in the presumptive fin field appear normal, the pectoral fin precursor cells display compaction and migration defects between 18 and 24 hours post-fertilization (hpf). During fin growth *fgf24 *is sequentially expressed in the mesenchyme and then in the apical ectodermal ridge (AER). However, in *pdlim7 *antisense morpholino-treated embryos this switch of expression is prevented and *fgf24 *remains ectopically active in the mesenchymal cells. Along with the lack of *fgf24 *in the AER, other critical factors including *fgf8 *are reduced, suggesting signaling problems to the underlying mesenchyme. As a consequence of perturbed AER function in the absence of Pdlim7, pathway components in the fin mesenchyme are misregulated or absent, indicating a breakdown of the Fgf signaling feedback loop, which is ultimately responsible for the loss of fin outgrowth.

**Conclusion:**

This work provides the first evidence for the involvement of Pdlim7 in pectoral fin development. Proper fin outgrowth requires *fgf24 *downregulation in the fin mesenchyme with subsequent activation in the AER, and Pdlim7 appears to regulate this transition, potentially through Tbx5 regulation. By controlling Tbx5 subcellular localization and transcriptional activity and possibly additional yet unknown means, Pdlim7 is required for proper development of the heart and the fins. These new regulatory mechanisms may have important implications how we interpret Tbx5 function in congenital hand/heart syndromes in humans.

## Background

The basic morphological and genetic mechanisms underlying vertebrate limb formation are highly conserved, from pectoral and pelvic fins in fish to arms and legs in humans [1-3]. Along the flank of the embryo the primordial limb fields are established at specific sites in the lateral plate mesoderm (LPM) [1]. The limb first appears as an outgrowth of mesenchyme from the LPM, which is covered by a sheet of ectoderm. The distal ectoderm covering the limb mesenchyme specializes and thickens to form a transient structure called the apical ectodermal ridge (AER). Reciprocal communication between the AER and underlying mesenchyme promotes cell proliferation and limb outgrowth. Physical removal of the AER, for example in the chicken embryo, results in cessation of limb growth and truncation of distal elements [4,5].

In all vertebrates, one of the earliest determinants defining the forelimb field is the T-box transcription factor Tbx5 [6-10]. Expressed in the limb mesenchyme, Tbx5 is required for forelimb development and its functional disruption in zebrafish, chicken, and mouse results in a complete loss of the limb [11-13]. Tbx5 transcriptionally activates *Fgf10 *in the forelimb mesenchyme [12], and its secreted gene product then signals to the AER to induce the expression of ectodermal Fgfs such as *Fgf4 *and *Fgf8 *[14-16]. Fgf8 in turn signals back to the underlying mesenchyme to maintain *Fgf10 *expression, thereby creating a feedback loop needed to support limb outgrowth and establish the proximal-distal limb axis.

The Fgf signaling pathway is critical for limb initiation and outgrowth [17]. Genetic disruption of *Fgf10*, *Fgf8*, or *Fgf4*/*Fgf8 *results in severely malformed or truncated limbs [15,16,18-21]. The secreted Fgf ligands can bind to four Fgf receptors (Fgfr), with Fgfr1 and Fgfr2 being essential for limb development [17]. *Fgfr1 *is expressed in the limb mesenchyme and is required for distal limb and digit formation [22,23]. *Fgfr2 *in the mouse is alternatively spliced into two isoforms, *Fgfr2b *and *Fgfr2c*, which are expressed in the ectoderm and mesenchyme, respectively [24]. Knock-out of both *Fgfr2 *isoforms results in a failure of limb induction [25,26] and deletion of isoform *Fgfr2b *causes limb defects due to a loss of AER maintenance [27,28]. While critical for proximal-distal limb patterning, Fgf signaling is also an integral part of patterning the other limb axes. The expression of *Shh*, a central signal in anterior-posterior axis patterning, as well as Wnts and Bmps, which participate in dorso-ventral axis formation, are all dependent on Fgf signals from the AER (reviewed in [29]).

We previously identified a member of the PDZ-LIM protein family, Pdlim7, to be co-expressed with and bind to the transcription factors Tbx5 and Tbx4 [30]. PDZ-LIM proteins contain an N-terminal PDZ domain and one or three C-terminal LIM domains. PDZ and LIM domains are both protein interaction modules, providing this multi-domain protein family with diverse interaction opportunities [31]. Functional roles for PDZ-LIM proteins have been reported in signal transduction, cell migration, and differentiation [32-35,31]. In cell cultures and chicken and zebrafish embryos we have shown that Pdlim7 regulates Tbx5 nuclear/actin cytoskeleton-associated localization and activity during cardiac atrioventricular boundary and valve formation [36-38]. Work in zebrafish revealed that Pdlim7 is also required for proper skeletal muscle development and maintenance [38,31]. However, the common or distinct functional roles Pdlim7 and related PDZ-LIM proteins have in organ formation in the developing vertebrate embryo remain poorly understood.

In the zebrafish, *pdlim7 *is co-expressed with *tbx5 *during cardiac and pectoral fin development [38]. Loss of either Pdlim7 or Tbx5 function leads to a similar cardiac phenotype, a non-looped heart, although by opposing molecular mechanisms [38,11]. Elimination of Tbx5 results in a loss of Tbx5 responsive gene activation while reduction of Pdlim7 leads to an upregulation of Tbx5 target genes at the atrio-ventricular boundary. Along with the heart problems observed at later developmental stages, compromised Pdlim7 activity also results in pectoral fin defects early in embryogenesis [38]. However, detailed analysis of the fin phenotype has not been performed. Here we analyze the pectoral fin phenotype induced by morpholino knock-down and mRNA overexpression of *pdlim7 *and provide the first evidence of a critical role for PDZ-LIM proteins in vertebrate limb development.

## Results

### Pdlim7 is required for pectoral fin development

**Figure 1:**
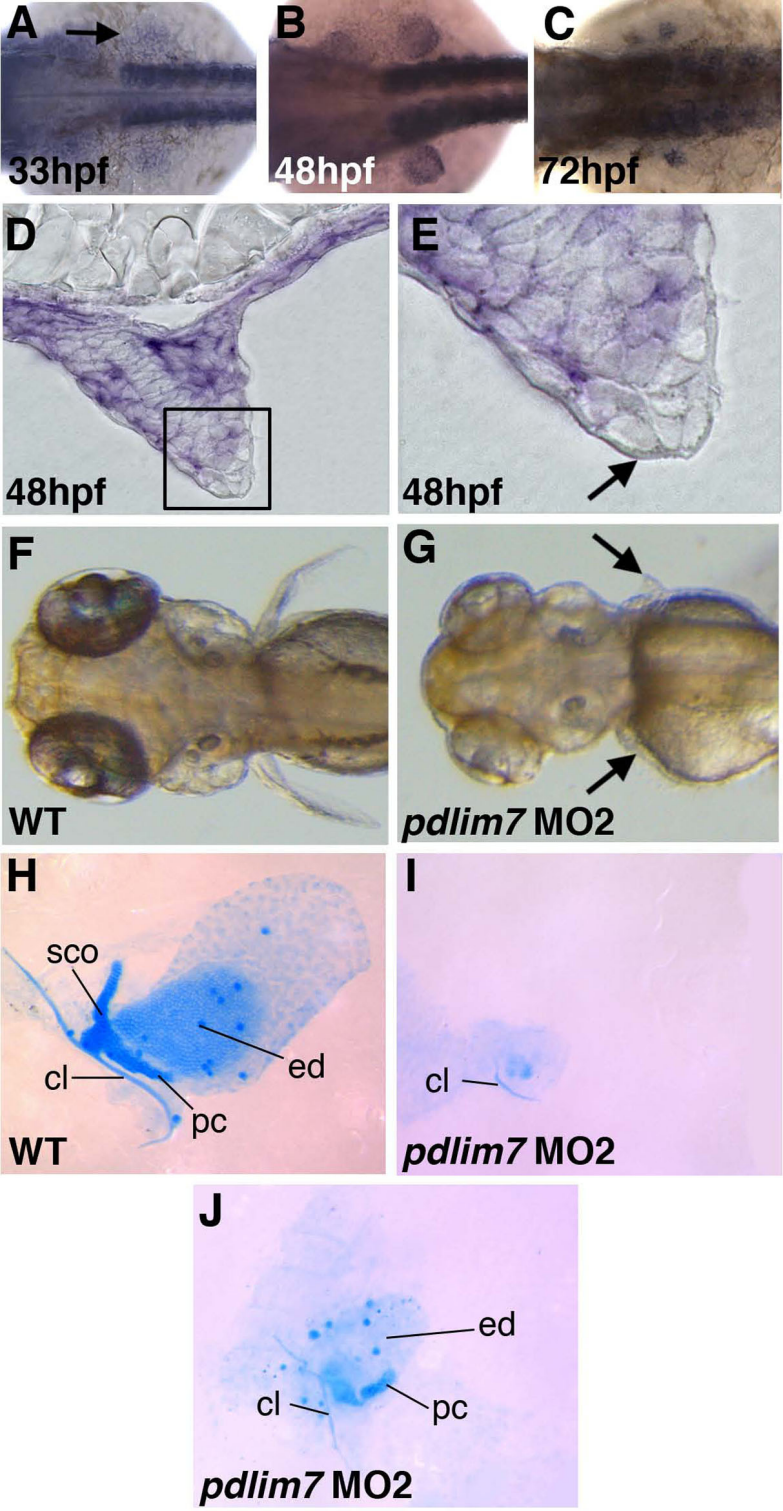
**Pdlim7 is required for pectoral fin development**. A-C: Whole-mount in situ hybridization using antisense RNA to show expression of *pdlim7 *in the developing pectoral fin mesenchyme at 33 hpf (A), 48 hpf (B), and 72 hpf (C). D-E: Sectioned embryos at 48 hpf of whole-mount in situ hybridization of *pdlim7 *show expression in fin mesenchyme. Boxed region in D is magnified in E to distinguish mesenchyme (purple color) and AER (arrow). F-G: Dorsal view of wild-type (F) and MO2 injected (G) embryos at 96 hpf. Arrows in G point to position of pectoral fin. H-J: Alcian blue stained cartilage preparations of dissected pectoral fins at 96 hpf. Wild-type (H), severe MO2 phenotype (I), and mild MO2 phenotype (J). I and J from same embryo. cl, cleithrum; pc, postcoracoid process; ed, endodermal disc; sco, scapulocoracoid.

The PDZ-LIM protein, Pdlim7, was identified as a novel binding protein and regulator of the transcription factor Tbx5 [30,36,38]. *Pdlim7 *mRNA has been detected in several tissues of the vertebrate embryo including the limbs, heart, and skeletal muscle [30,36]. In the zebrafish embryo during fin development, using whole mount in situ hybridization, we first detected *pdlim7 *expression in the mesenchyme of the fin field at 33 hours post-fertilization (hpf) which was maintained in the fin up to 72 hpf (Fig. 1A-E; [38]). Expression of *pdlim7 *was not detected in the AER (Fig. 1D, E). Injection at the one-cell stage with 2 ng of *pdlim7 *antisense morpholino oligonucleotides, interfering with either protein translation (MO1) or RNA splicing (MO2), resulted in comparable defects in pectoral fin development (Fig. 1 and data not shown; see Methods). At 48 hpf, embryos injected with MO2 produced phenotypes with stunted fin buds as compared to control siblings, indicating a possible defect in forelimb outgrowth ([38]; data not shown). By four days of development in wild-type larvae the pectoral fins are clearly visible, while the pectoral fins of MO2 injected embryos were significantly smaller or absent (Fig. 1F-G). Both control and morphant larvae were stained with Alcian blue to visualize the extent of cartilage differentiation. In controls, all of the major cartilage elements were present in the pectoral fin (Fig. 1H). However, in *pdlim7 *MO2 injected larvae, cartilage development was severely impeded. In the majority of cases, only a fragment of the cleithrum bone along with limited unidentifiable cartilage condensation could be detected (Fig. 1I). Some phenotypic variability was observed among MO2 treated embryos and occasionally slight differences in phenotype were visualized between left and right pectoral fins within single embryos. Less severe pectoral fin phenotypes in morphant embryos resulted in larvae with elements of the cleithrum, postcoracoid process, and a greatly reduced endochondral disc (Fig. 1J). Based upon mRNA expression and gene knock-down data, Pdlim7 appears to be required for pectoral fin development.

### Cell proliferation is decreased in pectoral fins after Pdlim7 knock-down

**Figure 2:**
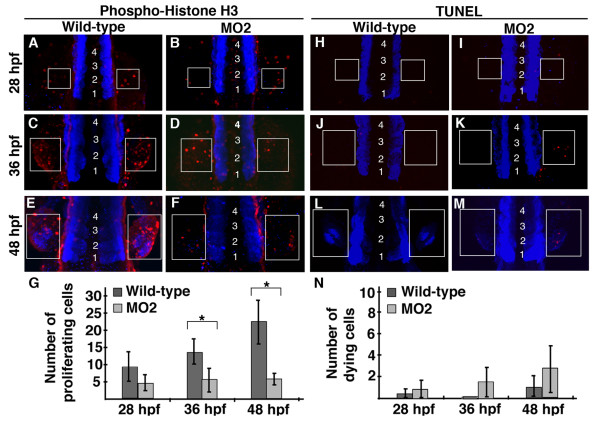
**Pdlim7 knock-down pectoral fins have decreased cell proliferation**. A-F: Dorsal view, anterior end of embryo is out of view to the bottom of image, of whole-mount anti-phospho-histone H3 (p-H3) antibody (red) staining on wild-type (A, C, E) and MO2 injected (B, D, F) embryos. Pectoral fins develop lateral to the 3^rd ^somite, thus embryos were counterstained with MF20 (blue) to visualize somites, which are indicated by numbers (somite 1 refers to the most anterior somite). G: Quantification of p-H3 positive cells in pectoral fins of wild-type and MO2 injected embryos. 28 hpf wild-type n = 5, MO2 n = 5; p-value = 0.077. 36 hpf wild-type n = 4, MO2 n = 5; p-value = 0.036. 48 hpf wild-type n = 5, MO2 n = 5; p-value = 0.001. Experiment performed in triplicate, representative data from single replicate shown in G. Statistically significant p-values (<0.05) are denoted by asterisks. H-M: Whole-mount TUNEL assay on wild-type (H, J, L) and MO2 injected (I, K, M) embryos. Apoptotic cells in red with MF20 stained somites in blue, as described for p-H3 staining. N: Quantification of apoptotic cells in wild-type and MO2 injected embryos. 28 hpf wild-type n = 6, MO2 n = 4; p-value = 0.483. 36 hpf wild-type n = 6, MO2 n = 5; p-value = 0.05. 48 hpf wild-type n = 4, MO2 n = 6; p-value = 0.073. Experiment performed in triplicate, representative data from single replicate shown in N. White boxes indicate pectoral fin field at 28 hpf (A-B, H-I), 36 hpf (C-D, J-K), and 48 hpf (E-F, L-M).

**Figure 3:**
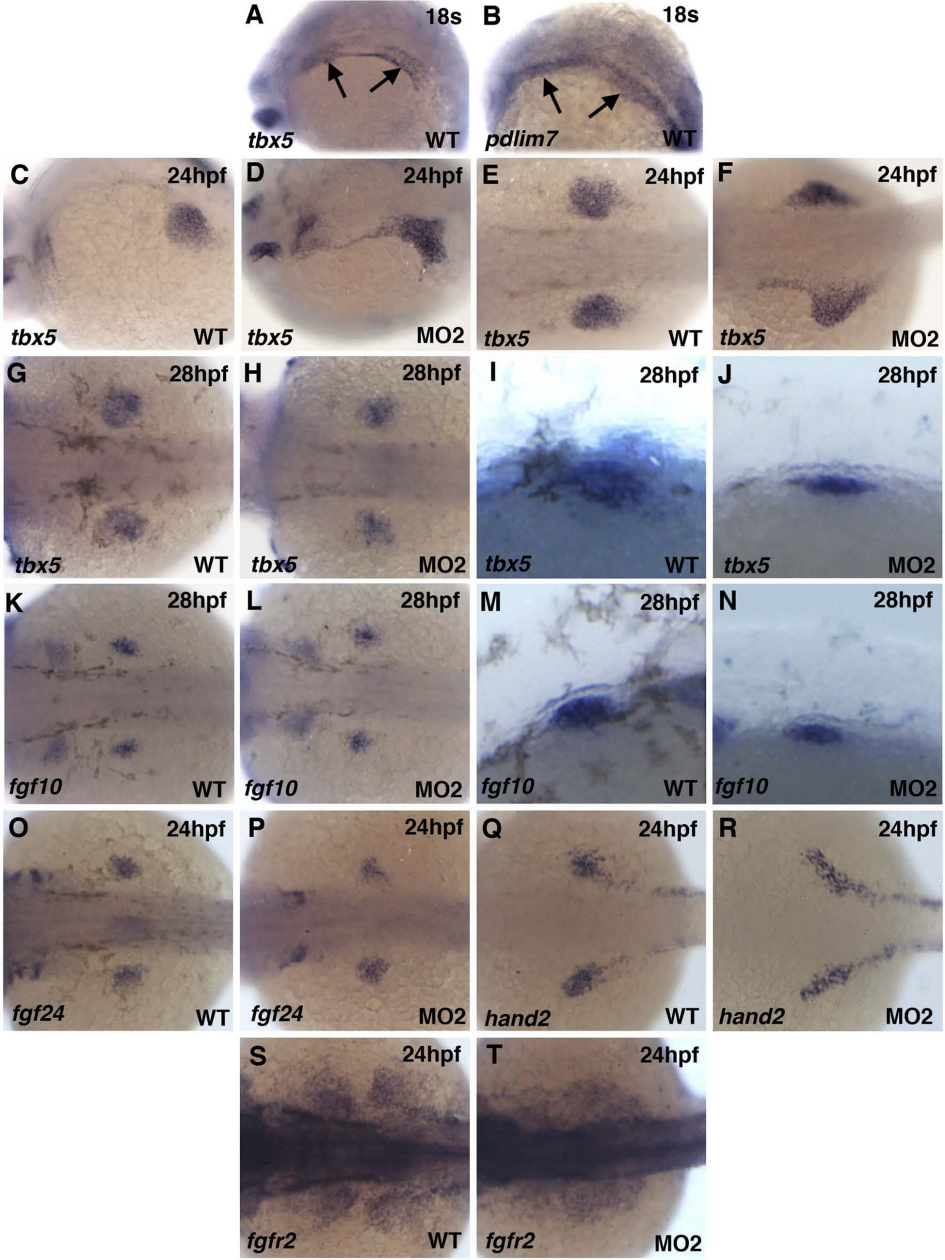
**Migration and compaction defects in *pdlim7 *MO2 injected embryos**. A-R: Whole-mount antisense RNA in situ hybridization of wild-type and MO2 injected embryos. *tbx5 *(A-H) expression at 24 hpf (A-D) and 28 hpf (E-H) in wild-type (A, C, E, G) and MO2 injected (B, D, F, H) embryos. G and H magnified lateral view of pectoral fin. *fgf10 *(I-L) expression at 28 hpf in wild-type (I, K) and MO2 injected (J, L) embryos. K and L magnified lateral view of pectoral fin. Expression at 24 hpf in wild-type and MO2 injected embryos of *fgf24 *(M-N), *hand2 *(O-P), and *fgfr2 *(Q-R), respectively. Head is positioned to the left.

**Figure 4:**
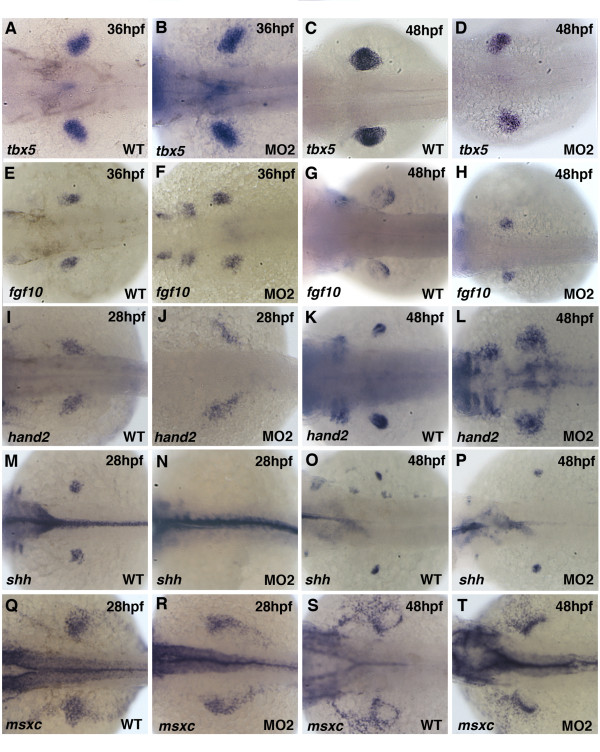
**Pectoral fin mesenchymal gene expression in Pdlim7 knock-down embryos**. Dorsal view of whole-mount antisense RNA in situ hybridization of wild-type (A, C, E, G, I, K, M, O, Q, S) and MO2 injected (B, D, F, H, J, L, N, P, R, T) embryos. A-D: *tbx5 *expression in wild-type and MO2 injected embryos at 36 hpf (A-B) and 48 hpf (C-D). E-H: *fgf10 *expression at 36 hpf (E-F) and 48 hpf (G-H). I-L: *hand2 *expression at 28 hpf (I-J) and 48 hpf (K-L). M-P: *shh *expression at 28 hpf (M-N) and 48 hpf (O-P). Q-T: *msxc *expression at 28 hpf (Q-R) and 48 hpf (S-T). Head is positioned to the left.

Knock-down of Pdlim7 results in the loss of, or severely truncated, pectoral fins. One possible cause for the fin phenotype could be due to alterations in cell proliferation or viability. To investigate this, embryos injected with *pdlim7 *MO2 were analyzed at stages of pectoral fin growth between 28 and 48 hpf for cell proliferation using an anti-phospho-histone H3 (p-H3) antibody or for cell death using TUNEL (Fig. 2; see Methods). Wild-type embryos displayed an increase in p-H3 antibody reactivity from 28, 36, to 48 hpf in the developing pectoral fin (Fig. 2A, C, E, boxed regions). At 28 hpf, in the pectoral fin field, MO2 injected embryos had a comparable number of dividing cells as wild-type (Fig. 2A, B). However, quantification of p-H3 positive cells revealed a steady increase of proliferating cells in wild-type pectoral fins as development progressed to 48 hpf, while cell proliferation remained at a low, constant level in *pdlim7 *knock-down embryos (Fig. 2D, F, G). Although the fin mesenchyme is smaller in morpholino injected embryos (see Figs. 3 and 4), the cells in the fin field including p-H3 positive cells appeared to be more scattered in the lateral plate mesoderm. Considering this, we used equal sized boxed regions in wild-type and MO2-treated embryos for analysis, which provides for a certain overestimation of dividing cells in morphant fins. Even with this conservative measure, we were able to detect a significant difference in p-H3 positive cells at 36 and 48 hpf between wild-type siblings and *pdlim7 *MO2 injected embryos (asterisks Fig. 2G; Additional file 1, Table S1).

One possible explanation for lower numbers of p-H3 positive cells in the pectoral fins may be reduced cell survival after *pdlim7 *knock-down. Therefore, using a TUNEL assay, we investigated apoptosis in wild-type and *pdlim7 *MO2 injected pectoral fins at 28, 36, and 48 hpf (Fig. 2H-M). In wild-type embryos, at most only one to two apoptotic cells could be observed in the developing pectoral fin at any of the three time points tested (Fig. 2H, J, L). In MO2 injected embryos, a slight increase of apoptotic cells in pectoral fins was detected, especially at 48 hpf (Fig. 2I, K, M). However, quantification of the data revealed that the slight increase in apoptotic cells in *pdlim7 *morphants was suggestive but not statistically significant compared to wild-type pectoral fins (Fig. 2N; Additional file 1, Table S1). These findings indicate that loss of Pdlim7 has no significant effect on apoptosis; however, the protein appears necessary for normal cell proliferation in the pectoral fin field.

### Pdlim7 knock-down embryos exhibit pectoral fin cell migration and compaction defects

Knock-down of Pdlim7 function leads to severe arrest in pectoral fin development and lower numbers of proliferating cells in the budding fin. We next sought to determine if the pectoral fin field was established correctly in the absence of *pdlim7*. In previous work we have demonstrated that Pdlim7 can regulate Tbx5 activity, one of the forelimb/pectoral fin field markers essential for limb outgrowth [36,38,8,10]. The cells of the fin field are derived from a population of cells in the LPM that initially comprises both heart and pectoral fin precursors [39]. The *tbx5 *expressing heart and pectoral fin progenitor cells remain indistinguishable until the 18-somite stage, when the pectoral fin precursor cells migrate posteriorly and separate from the adjacent anterior cardiac progenitors. The migratory behavior of these cells has been shown to be dependent upon Tbx5 activity [39]. Of note, in wild-type embryos both fin and heart precursor cells co-express *tbx5 *and *pdlim7 *(Fig. 3A, B), and by 24 hpf, *tbx5 *expressing cells are completely separated into the heart and pectoral fin primordia (Fig. 3C). In contrast, *tbx5 *expressing cells in 24 hpf *pdlim7 *morphants were detected in the LPM between the heart tube and pectoral fin field, connecting the two organ fields (Fig. 3D). Dorsal views of MO2 injected embryos also displayed a less compact pectoral fin field as visualized by *tbx5 *expression (Fig. 3E, F). In comparison to control embryos, at 28 hpf, the pectoral fin field was noticeably smaller in size and induction of limb outgrowth appeared defective (Fig. 3G-J). These results suggested aberrant or delayed cell migration of forelimb precursors into the fin field, although overall embryonic development did not appear significantly delayed in *pdlim7 *morphants as cardiac beating began as expected around 22 hpf.

The Tbx5 downstream target gene, *fgf10*, is expressed in the pectoral mesenchyme at 28 hpf; right at the time when the fin bud emerges from the LPM (Fig. 3K, M; [40]). Although fin bud growth was disrupted, *fgf10 *was induced normally in *pdlim7 *knock-down embryos (Fig. 3K-N), suggesting that the Pdlim7 mediated misregulation of Tbx5 transcriptional activity upon *fgf10 *may not be the cause of the compromised fin outgrowth.

To further investigate whether the pectoral fin primordium was fully established, we analyzed the expression of the early specification markers *fgf24*, *hand2*, and *fgfr2*. All of these genes are active in the LPM in the fin primordial cells [41-43]. Fgf24 is expressed in the pectoral fin precursor mesenchyme and is functionally required for cell migration and compaction to the presumptive fin field [41]. At 24 hpf in control embryos, *fgf24 *clearly delineated the future location of the pectoral fins (Fig. 3O). *fgf24 *expression was detected after *pdlim7 *knock-down, although the expression domain was slightly smaller and appeared closer to the midline of the embryo (Fig. 3P). *hand2 *was detected in the presumptive fin mesenchyme by 24 hpf in control and MO2 injected embryos (Fig. 3Q-R; [42]). However, in morphant embryos, *hand2 *expression failed to undergo mediolateral expansion in the LPM. Expression of *fgfr2*, which is thought to be downstream of Tbx5 [43], was detected in the compact primordial pectoral fin cells in wild-type embryos (Fig. 3S). In the morphants, *fgfr2 *expression was diffuse and did not display normal compaction (Fig. 3T), similar to the compaction defect of *tbx5 *expressing cells (Fig. 3F). In *pdlim7 *knock-down embryos, mesenchymal gene induction and pectoral fin cell specification appears to occur normally, however, early compaction of the primordial fin field is disrupted or delayed possibly due to incomplete precursor cell migration.

### Knock-down of Pdlim7 does not disrupt pectoral fin mesenchyme patterning

Molecular markers for early pectoral fin specification were normal in *pdlim7 *compromised embryos, although precursor cell migration appeared delayed and the resulting fin field was smaller and less compact. In order to gain a better understanding of the cause of the pectoral fin phenotype, we examined the expression of several mesenchymal markers involved in limb patterning. We first analyzed *tbx5*, which is regulated by Pdlim7 in the zebrafish heart [38]. By 36 and 48 hpf, *tbx5 *expression could be detected in wild-type embryos throughout the developing pectoral fin mesenchyme (Fig. 4A, C; [11]). In *pdlim7 *knock-down embryos, *tbx5 *expression was maintained in the fin mesenchyme, however, the expression domain appeared less compact when compared to controls (Fig. 4A-D). *Fgf10 *is a direct downstream target of Tbx5 in the forelimb [12,13,40]. As expected, in wild-type and *pdlim7 *MO2 injected embryos at both 36 and 48 hpf, *fgf10 *was expressed in the limb mesenchyme; however, at the later time point the *fgf10 *expression domain appeared smaller in the morphants and less concentrated to the distal mesenchyme (Fig. 4E-H). The detection of *fgf10 *in the pectoral fin mesenchyme of MO2 treated embryos suggested that Tbx5 protein was transcriptionally functional and proximal-distal limb outgrowth had been initiated.

We next tested the expression of *hand2 *and *shh*, two genes required for limb development and involved in anterior-posterior patterning [44,42,46]. *hand2 *is normally expressed in the posterior mesenchyme of the developing pectoral fin (Fig. 4I, K; [42]). In *pdlim7 *MO2 injected embryos, *hand2 *expression was detected in the LPM between 28 and 48 hpf, though its expression was diffuse and covered a wider area as compared to the control embryos (Fig. 4I-L; data not shown). Similar to *hand2*, *shh *is asymmetrically expressed in a posterior mesenchymal domain of the developing fin (Fig. 4M, O; [47]). At 28 hpf, in wild-type embryos, *shh *revealed this posterior expression, however, in the MO2 injected embryos no expression could be detected in the pectoral fin (Fig. 4M, N). Of note, *shh *expression was observed in the floor plate of morphant embryos at the same 28 hpf time-point (Fig. 4N; data not shown). Expression of *shh *recovered in the pectoral fin by 48 hpf, suggesting the absence at 28 hpf may have been due to a delay in activation (Fig. 4O, P). Despite the significant reduction in fin size at 48 hpf, *shh *remained asymmetrically expressed in a posterior domain in the *pdlim7 *knock-down embryos (data not shown). Another gene involved in patterning all three limb axes is *msxc *[48]. At 28 hpf, *msxc *was expressed in the pectoral fin mesenchyme in both control and *pdlim7 *morphant embryos (Fig. 4Q-R; [49]). Similar to the other mesenchymal markers tested, *msxc *displayed a very diffuse pectoral fin expression at 36 and 48 hpf in MO2 injected embryos compared to the controls (Fig. 4S-T; data not shown). The localization of *msxc *in the mesenchyme adjacent to the AER appeared to recover by the later time-point, however, some *msxc *expressing cells remained in the LPM (Fig. 4T). Most of the key regulators tested in the pectoral fin mesenchyme remained expressed in *pdlim7 *knock-down embryos, however, the overall size and domain organization in the fin field appeared significantly affected.

### Pdlim7 knock-down causes loss of AER gene expression

**Figure 5:**
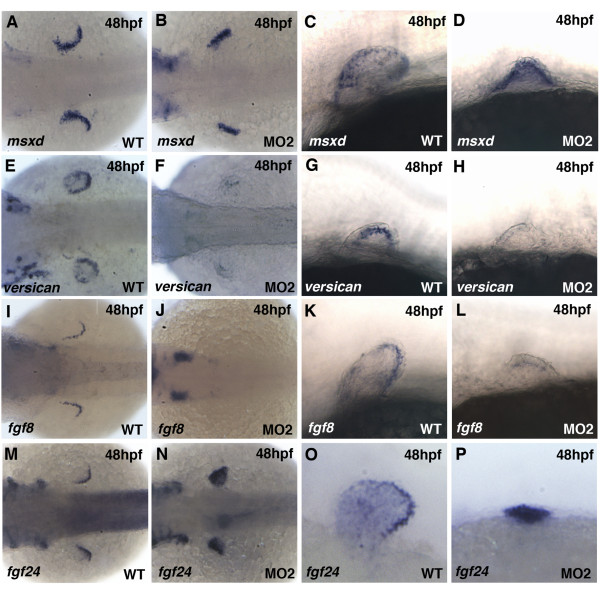
**Disruption of AER gene expression in *pdlim7 *MO2 injected embryos**. A-D: Expression of *msxd *at 48 hpf in wild-type (A, C) and MO2 injected (B, D) embryos. E-H: Expression of *versican *in wild-type (E, G) and MO2 injected (F, H) embryos. I-L: Expression of *fgf8 *in wild-type (I, K) and MO2 injected (J, L) embryos. M-P: Expression of *fgf24 *in wild-type (M, O) and MO2 injected (N, P) embryos. Dorsal views (A, B, E, F, I, J, M, N) and lateral views (C, D, G, H, K, L, O, P). Head is positioned to the left.

Knock-down of Pdlim7 results in a stunted fin phenotype (Fig. 1; [38]), suggesting a defect in limb outgrowth. Limb outgrowth requires continuous signaling between the distal AER and the underlying limb mesenchyme [1,2,29]. Since gene activities involved in patterning the pectoral fin mesenchyme were detected in the morphant embryos, we next asked whether gene expression in the AER was perturbed in Pdlim7 compromised embryos. At 48 hpf we detected expression of the fin AER marker *msxd *[49] in wild-type and *pdlim7 *MO2 injected embryos alike, indicating that the AER had formed (Fig. 5A-D). The proteoglycan *versican *was also detected in the AER of developing pectoral fins of wild-type embryos (Fig. 5E, G). We have found *versican *to be misregulated in the heart of *pdlim7 *MO2 injected embryos [38], and therefore wondered whether *versican *would be misexpressed in the pectoral fins as well. Indeed, *versican *was either greatly reduced or absent from the pectoral fins in morphant embryos (Fig. 5F, H). Another prominent AER marker gene is *fgf8*, whose function is required to maintain the signaling loop between the AER and underlying limb mesenchyme for appendage outgrowth [19,50,20]. Knock-down of Pdlim7 resulted in a significant reduction of *fgf8*, compared to sibling controls (Fig. 5I-L). The family member *fgf24 *is normally turned off in the fin mesenchyme by 48 hpf and subsequently expressed in the overlying AER (Fig. 5M, O; [41]). Importantly, unlike in the control embryos, we could not detect the switch from mesenchymal to AER expression for *fgf24 *after *pdlim7 *knock-down, but we rather observed ectopic expression in the fin mesenchyme (Fig. 5N, P). Therefore, the loss of fin outgrowth in *pdlim7 *morphant embryos appears to be due to a loss of proper AER function and disruption of the reciprocal signaling between AER and the adjacent mesenchyme.

### Knock-down of pdlim7 affects Fgf signaling in the pectoral fin

**Figure 6:**
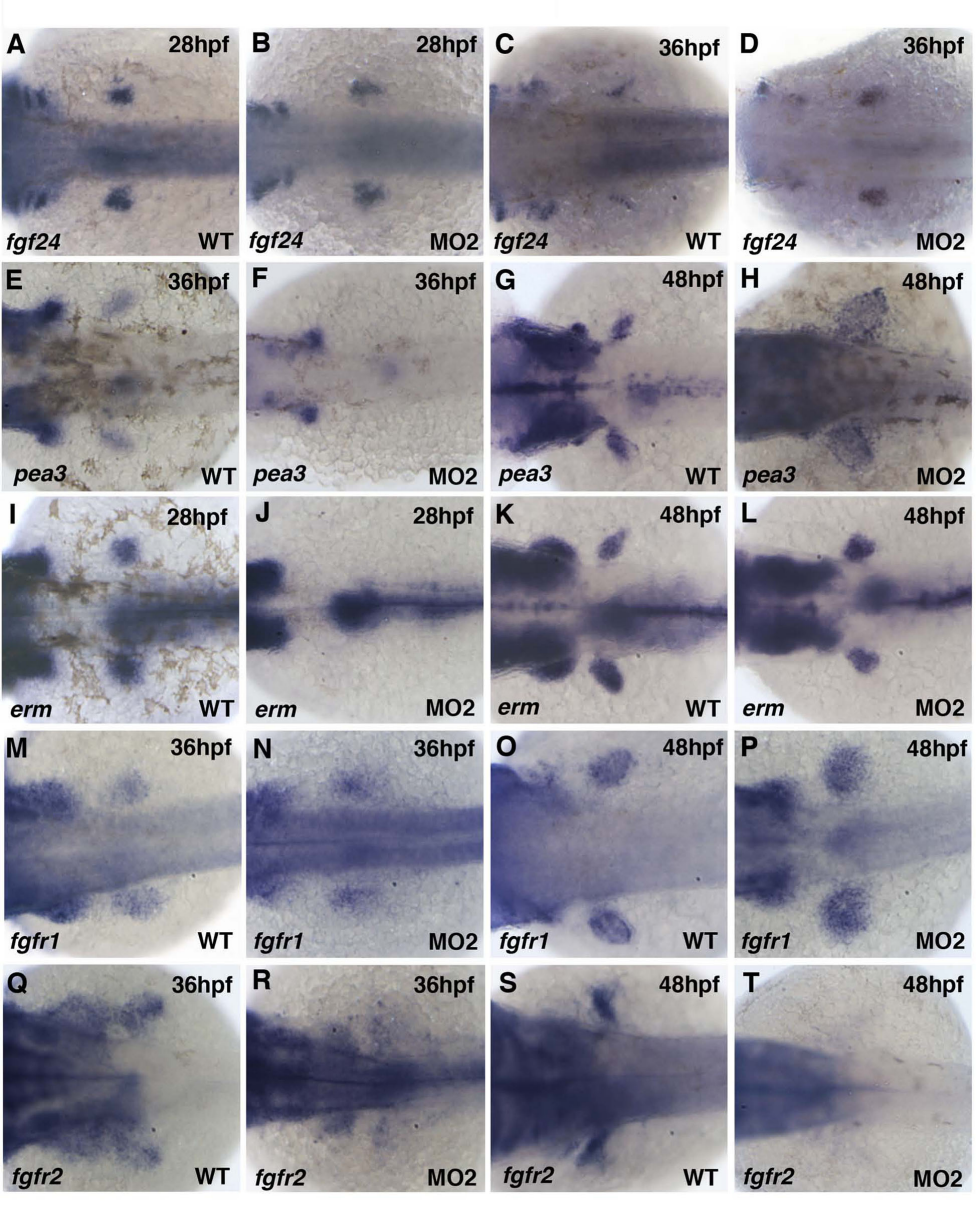
**Fgf signaling pathway genes are disrupted after knock-down of Pdlim7**. Dorsal view of whole-mount antisense RNA in situ hybridization of wild-type (A, C, E, G, I, K, M, O, Q, S) and MO2 injected (B, D, F, H, J, L, N, P, R, T) embryos. A-D: Expression of *fgf24 *at 28 hpf (A-B) and 36 hpf (C-D). E-H: Expression of *pea3 *at 36 hpf (E-F) and 48 hpf (G-H). I-L: Expression of *erm *at 28 hpf (I-J) and 48 hpf (K-L). M-P: Expression of *fgfr1 *at 36 hpf (M-N) and 48 hpf (O-P). Q-T: Expression of *fgfr2 *at 36 hpf (Q-R) and 48 hpf (S-T). Head is positioned to the left.

The disruption and loss of Fgf gene expression in the AER of *pdlim7 *MO2 injected embryos suggested a possible breakdown of the Fgf signaling loop between the mesenchyme and AER. To further explore this possibility, we tested additional Fgf signaling components in the pectoral fin after Pdlim7 protein reduction. Fgf24 is a zebrafish-specific factor that functions downstream of Tbx5 in pectoral fins and is required to activate *fgf10 *expression in the fin mesenchyme [41]. At 24 and 28 hpf, *fgf24 *was detected in the pectoral fin mesenchyme of wild-type embryos (Figs. 3O and 6A) and did not appear significantly different in embryos injected with *pdlim7 *MO2 (Figs. 3P and 6B) or earlier time-points (Fig. 5M, N). In contrast, in 36 hpf *pdlim7 *compromised embryos, *fgf24 *expression remained in the fin mesenchyme and did not switch to the AER (Fig. 6C-D). Ectopic expression of *fgf24 *in the *pdlim7 *morphant mesenchyme was maintained at 48 hpf with no detectable expression in the AER (Fig. 5M-P). We consequently tested two transcriptional targets of Fgf signaling in the fin mesenchyme, *pea3 *and *erm *[51,52]. We could detect *pea3 *expression in the pectoral fins at 36 and 48 hpf of control embryos (Fig. 6E, G). However, in 36 hpf morphant embryos, *pea3 *mRNA was absent from the LPM but appeared to recover by 48 hpf (Fig. 6F, H). Despite the presence of *pea3 *expression at 48 hpf, its localization was greatly diffused in the fin and did not show the normal restriction within the mesenchyme (Fig. 6G, H). A similar result was also observed for *erm*, a gene normally expressed in the budding pectoral fin (Fig. 6I, K). At 28 hpf in MO2 injected embryos, we could not detect *erm *mRNA in the early bud, but gene expression recovered by 48 hpf, although with a slightly smaller and irregular shaped domain than in controls (Fig. 6I-L).

Sandwiched between the secreted Fgf signaling molecules and downstream target genes are the Fgf receptors, whose function is critical for signal transduction and limb formation [17]. We tested the expression of *fgfr1 *and *fgfr2 *in the developing pectoral fins of control and *pdlim7 *morphant embryos. At 36 and 48 hpf, *fgfr1 *was expressed in wild-type pectoral fins throughout the developing mesenchyme (Fig. 6M, O). In MO2 injected embryos; however, similar to other genes tested, *fgfr1 *displayed a loss of compaction (Fig. 6M-P). *fgfr2 *mRNA was also detected in the fin mesenchyme of control embryos at the two time-points, with expression restricted by 48 hpf to the proximal and anterior portion of the pectoral fin (Fig. 6Q, S). After knock-down of Pdlim7, *fgfr2 *levels were significantly reduced at 36 hpf and expression completely absent from the pectoral fin at 48 hpf (Fig. 6R, T). Thus, there appears to be a gradual loss of *fgfr2 *function between 24 and 48 hpf (Fig. 3 and Fig. 6). Of note, expression of *fgfr2 *in the head and brain was not affected by injection of *pdlim7 *MO2 (data not shown). Taken together, these results reveal that, after *pdlim7 *knock-down, the expression of several components of the Fgf signaling pathway known to be critical for limb outgrowth, including the AER/mesenchyme signaling loop, are misregulated or absent.

### Pdim7 overexpression does not alter mesenchymal Fgf expression

**Figure 7:**
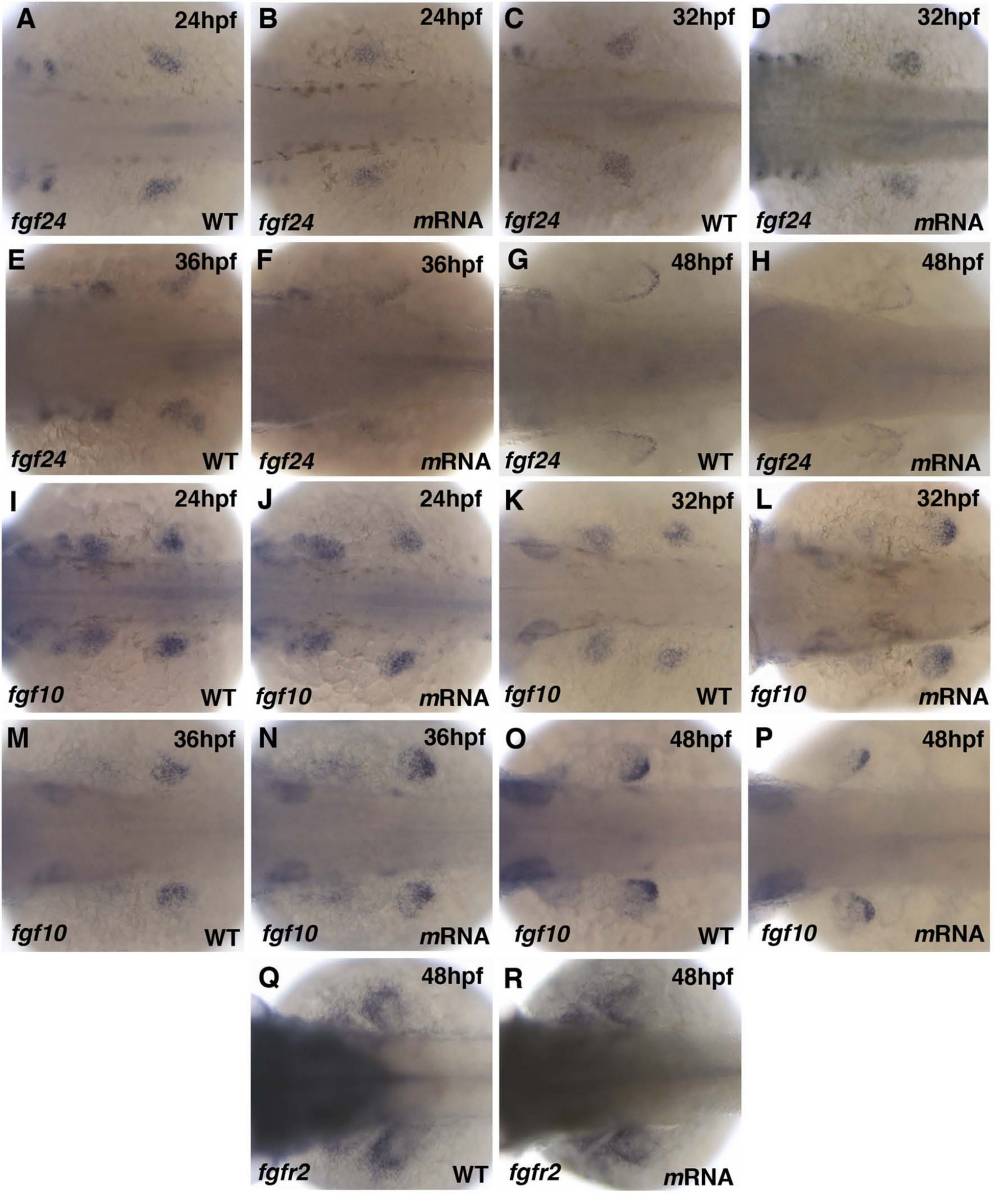
**Pdlim7 overexpression does not alter Fgf signaling genes**. Dorsal view of whole-mount antisense RNA in situ hybridization of wild-type (A, C, E, G, I, K, M, O, Q) and 100 pg synthetic *pdlim7 *mRNA injected (B, D, F, H, J, L, N, P, R) embryos. A-H: Expression of *fgf24 *at 24 hpf (A-B), 32 hpf (C-D), 36 hpf (E-F), and 48 hpf (G-H). I-P: Expression of *fgf10 *at 24 hpf (I-J), 32 hpf (K-L), 36 hpf (M-N), and 48 hpf (O-P). Q-R: Expression of *fgfr2 *at 48 hpf.

The ectopic expression of *fgf24 *in the fins of *pdlim7 *morpholino injected embryos suggested that *fgf24 *maybe a direct target of Tbx5 and subject to regulation by Pdlim7. In an effort to support this hypothesis and to complement the knock-down studies, we overexpressed *pdlim7*. Considering Pdlim7 induced shuttling of Tbx5, injection of 100 pg *pdlim7 *mRNA into one-cell stage embryos should sequester Tbx5 from the nucleus, resulting in the downregulation of target genes. Comparison of in situ hybridization for *fgf24 *between wild-type and mRNA injected embryos at 24, 32, 36, and 48 hpf displayed no significant changes in expression (Fig. 7A-H). Expression of *fgf24 *during the fin progenitor cell compaction appeared normal at 24 hpf (Fig. 7A, B) while the gene still transitioned from mesenchymal to strict AER localization between 32 and 48 hpf (Fig. 7C-H). In addition to *fgf24*, we monitored expression of *fgf10 *after *pdlim7 *overexpression. *fgf10 *did not reveal significant differences at 24, 32, 36, or 48 hpf between uninjected control siblings and *pdlim7 *mRNA injected embryos (Fig. 7I-P). *fgf10 *revealed its typical dynamic expression from broad mesenchymal (Fig. 7I-N) to concentrated location in the distal mesenchyme adjacent to the AER by 48 hpf (Fig. 7O, P). In several mRNA injected embryos we noticed a more pronounced *fgf10 *crescent shape expression domain adjacent to the AER, which is typical for a more mature limb and may indicate slightly accelerated differentiation caused by forced *pdlim7 *expression. Finally, since *fgfr2 *was greatly affected after *pdlim7 *knock-down and significantly downregulated by 48 hpf (Fig. 6), we determined the expression of *fgfr2 *after *pdlim7 *overexpression. Similar to the observations for *fgf24 *and *fgf10*, *fgfr2 *at 48 hpf did not display differential expression between wild-type versus the mRNA injected embryos and stained the proximal portion of the fin bud (Fig. 7Q, R). Control in situ hybridizations for the Tbx5 target gene *tbx2b *revealed, however, a reduction of expression at the heart AV boundary as previously published (data not shown and [38]), demonstrating that the injected synthetic mRNA was functional. The lack of change in *fgf24 *or *fgf10 *expression after *pdlim7 *overexpression does not rule out that *fgf24 *is directly activated by Tbx5 or regulated by Pdlim7, as maintenance of limb outgrowth and *Fgf10 *expression in the mouse is independent of Tbx5 [53].

## Discussion

### Pdlim7 functions to regulate Tbx5 transcriptional activity

**Figure 8:**
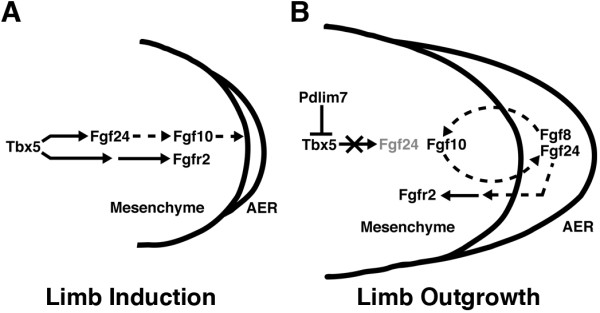
**Model for vertebrate limb induction and outgrowth**. During zebrafish fin induction, Tbx5 activates parallel pathways in the lateral plate mesoderm. In one pathway, Tbx5 transcriptionally activiates *fgf24*, which then initiates a signaling cascade leading to the activation of *fgf10*. In a second pathway, Tbx5 activity indirectly regulates the expression of *fgfr2 *(A). During fin outgrowth, Tbx5 transcriptional activity is negatively regulated by Pdlim7-mediated nucleocytoplasmic shuttling. Relocation and retention of Tbx5 at the actin cytoskeleton results in transcriptional restriction of *fgf24 *in the limb mesenchyme. The mesenchymal downregulation is required for initiating Fgf24 expression in the AER, which is a critical step to establish the reciprocal signaling loop with the mesenchyme to maintain *fgf10 *and *fgfr2 *expression (B). Solid arrows denote transcriptional regulation. Dashed arrows indicate signal transduction regulation.

T-box proteins including Tbx5 contain nuclear localization and nuclear export sequences, enabling these transcription factors to relocate between nuclear and cytoplasmic cell compartments [54-56]. We have previously shown that Pdlim7 is necessary for dynamic shuttling of Tbx5, sequestering the transcription factor to actin filaments outside the nucleus and thereby regulating Tbx5 target gene expression both in vitro and in vivo [30,36,38]. For example, *tbx2b *and *nppa *are downstream targets of Tbx5 during zebrafish heart valve development and can be indirectly regulated by Pdlim7 levels in cells of the atrio-ventricular (AV) boundary [38]. Loss of Pdlim7 function by morpholino knock-down results in increased *tbx2b *and *nppa *expression with excess valve tissue, while overexpression of Pdlim7 by synthetic mRNA injection into the embryo causes downregulation of these Tbx5 target genes and reduced valve tissue. These experimental results lead to a model in which a balance of Pdlim7 and Tbx5 within the cell regulates transcription factor activity. Does a similar molecular mechanism function during forelimb development? *Fgf10 *has been shown to be a transcriptional target of Tbx5 using in vitro reporter assays and in vivo during mouse forelimb development [12]. In addition, in cultured cells, Pdlim7 can regulate Tbx5 activation of an Fgf10-luciferase reporter construct [36]. Work from this study in the developing zebrafish pectoral fins, however, suggests that misregulation of Pdlim7 does not cause significant changes in *fgf10 *expression. Knock-down of *pdlim7 *did not result in an obvious upregulation or ectopic expression of *fgf10 *and respective overexpression of Pdlim7 did not lead to an apparent decrease in *fgf10 *expression (Fig. 3, 4, 7), however, the smaller fin size in the morphant embryos may obscure a correct assessment and contribute to this observation. Interestingly, the zebrafish specific Fgf24 has been placed in a pathway between Tbx5 and Fgf10 during pectoral fin induction (Fig. 8A; [41,43]). *fgf24 *is expressed in the presumptive pectoral fin cells in the LPM at 18 hpf and is maintained in the fin mesenchyme until 28 hpf, before the gene is downregulated and then activated in the AER by 32 hpf (Fig. 6 and 7; [41]). Of note, we could detect induction of *pdlim7 *mRNA by 32 hpf in the budding fin, the time point when *fgf24 *mesenchymal expression is turned off. The switch between mesenchymal to AER expression of *fgf24 *does not occur in *pdlim7 *knock-down embryos; however, we could demonstrate ectopic expression of *fgf24 *in the fin mesenchyme up to 48 hpf (Fig. 5 and Fig. 6). The fact that Pdlim7 function can influence *fgf24 *expression in the fin mesenchyme, coupled with previous findings that loss of Tbx5 results in a loss of *fgf24 *expression [41], suggests that Fgf24 may be the critical Fgf target of Tbx5 in the zebrafish fin.

Based upon this suggestion we asked whether overexpression of *pdlim7 *would inhibit mesenchymal *fgf24 *expression before the normal appearance of *pdlim7*. *Pdlim7 *mRNA injection, however, did not yield an obvious reduction of *fgf24 *or *fgf10 *expression levels at 24 hpf in the fin mesenchyme, a time window before endogenous *pdlim7 *appears (Fig. 7A, B, I, J). This may be because expression of Pdlim7 and Tbx5 in a cell does not automatically result in binding and sequestration of the transcription factor. This notion is supported by co-expression of Pdlim7 and Tbx5 in undifferentiated cultured chicken epicardial cells that have strict nuclear localization of Tbx5, suggesting that the interaction of both proteins is regulated by yet unknown mechanisms, possibly posttranslational modifications [37]. Thus, early ectopic expression of *pdlim7 *may not be sufficient to result in failure of *fgf24 *expression. During the later stages (32-48 hpf) of limb outgrowth, *pdlim7 *overexpression also did not result in significantly perturbed *fgf24 *or *fgf10 *expression. The reason for this may lie in the possibility that Tbx5 is not required for maintenance of *fgf24 *and *fgf10 *expression. Recent experiments in the mouse have shown that Tbx5 is only required for induction of *Fgf10 *during limb initiation but not for maintenance during limb outgrowth [53]. If a similar regulation operates in the zebrafish, overexpression of Pdlim7 in the fin mesenchyme, during stages after 32 hpf when Tbx5 nuclear activity may not be critical, will have little to no affect on Tbx5 target gene expression. This could explain why *fgf24 *and *fgf10 *are maintained after *pdlim7 *mRNA injection. In contrast, loss of Pdlim7 may act as a gain-of-function with regard to Tbx5 activity resulting in ectopic *fgf24 *in the fin mesenchyme. Consistent with this hypothesis, the genomic sequence just upstream of the *fgf24 *gene contains a putative consensus Tbx5 binding element ([57], data not shown), which would provide a mechanism for *fgf24 *regulation by Pdlim7/Tbx5 interactions. Additional experiments at the genomic DNA level will be needed to fully resolve this point.

Knock-down of *pdlim7 *causes persistent high levels of nuclear Tbx5 and continued ectopic expression of *fgf24 *in mesenchymal fin cells, which likely accounts for *fgf10 *maintenance despite the loss of AER genes such as *fgf8*. The expression of *fgfr2*, which appears to be in a parallel pathway downstream of Tbx5 during fin induction [43], is induced normally in *pdlim7 *MO2 injected embryos but becomes lost at post-induction stages possibly due to the absence of reciprocal AER signaling. We hypothesize that the signal required in the AER to maintain *fgfr2 *expression during fin outgrowth is Fgf24. *fgf24 *is never activated in the AER of *pdlim7 *morphants and the downregulation of *fgfr2 *appears to begin when *fgf24 *is supposed to switch from mesenchymal to ectodermal expression. This new information adds to our understanding of vertebrate limb induction and outgrowth and allows the extension of current models (Fig. 8A, B). During limb induction Tbx5 operates in at least two feed-forward parallel pathways. In one branch of the pathway, Tbx5 activates transcription of *fgf24*, which then leads to the expression of *fgf10 *and signaling to the AER. The other branch regulates via a genetic cascade leading to *fgfr2 *activation (Fig. 8A). During limb outgrowth after 32 hpf, Pdlim7 in the fin mesenchymal cells control Tbx5 activity by removing the transcription factor from the nucleus (Fig 8B). As a consequence, *fgf24 *becomes down-regulated, allowing a switch from mesenchymal to AER expression. Fgf signaling molecules, including Fgf24 and Fgf8, emanating from the AER maintain *fgf10 *and *fgfr2 *expression in the underlying mesenchyme. The reciprocal signaling between the AER and mesenchyme becomes independent of Tbx5 activity during limb outgrowth (Fig. 8B; [53]). Although the new data presented here clearly demonstrate that appropriate regulation of Tbx5 via Pdlim7 is necessary for establishment of a functional AER through Fgf24 expression, future investigations confirming this hypothesis may reveal the mechanistic details for Pdlim7 regulating Tbx5 balance and transcriptional activity in the developing forelimbs.

### Pdlim7 in cell migration and compaction

Tbx5 has been shown to be required for proper pectoral fin cell migration during zebrafish development [39]. We also found evidence for defective cell migration in *pdlim7 *MO2 injected embryos (Fig. 3). *tbx5 *expressing pectoral fin cells display delayed migration and disrupted compaction of the fin field. This defect does not appear to be due to an overall developmental delay in *pdlim7 *morphants, as the heart begins to beat in a comparable time window as uninjected controls. A similar phenotype is also seen in *ikarus *(*ika*) zebrafish mutants, which lack *fgf24 *function [41]. In these mutant embryos, *tbx5 *expressing cells fail to undergo appropriate compaction at the pectoral fin field. The primordial fin cells at 18-somites express *tbx5*, *pdlim7 *as well as *fgf24 *(Fig. 3, [10,41]). Considering *fgf24 *is a target of Tbx5, it is possible that Pdlim7/Tbx5 protein interactions regulate *fgf24 *expression already in the 18-somite-stage embryo. Increased nuclear Tbx5, by loss of Pdlim7, would result in misregulation of *fgf24*. This idea is supported by the similar compaction defects observed after *pdlim7 *knock-down and in *fgf24 ika *mutants (Fig. 3; [41]). Overexpression as well as loss of Tbx5 causes defects in cell migration in cultured chicken proepicardial cells [58]. It is plausible that Pdlim7 functions at an initial stage of migration, in part by regulating Tbx5 balance in the nucleus and cytoplasm.

An alternative view would be that Pdlim7 itself, by its nature as an actin-associated protein, may modulate actin cytoskeleton dynamics and in this way directly influence cell migration [30,36]. In this context it is of interest that members of the PDZ-LIM protein family have been reported to be involved in cell migration [59-61]. However, more direct experimentation is required to fully elucidate this intriguing possibility.

### Pdlim7 may be involved in Fgf signal transduction

In zebrafish, knock-down of *pdlim7 *by morpholino injection results in severely stunted pectoral fins, decreased cell proliferation in the fin region, and a loss or altered expression of Fgf pathway genes. Fgf signal transduction from the AER is required for limb outgrowth and proliferation of the undifferentiated limb mesenchyme [17,62]. How might Pdlim7 be involved in Fgf signaling in the developing pectoral fin? The delayed migration of cells into the pectoral fin field, coupled with the reduced ability of the cells in the limb field to compact after loss of Pdlim7 function, may contribute to decreased Fgf signaling. The lower proliferation rate in the budding fins further equates to fewer cells secreting and responding to signaling molecules, lowering the signaling potential. Thus, it is possible that a certain minimum threshold needed for signal propagation is not reached and the mesenchyme/epithelial reciprocal interactions required for limb outgrowth are not established or maintained. This scenario may account for the maintenance of mesenchymal Fgfs but loss of Fgf expression in the AER.

A second possibility could be that Pdlim7 plays a more direct role in Fgf signal transduction. Pdlim7 can interact with transmembrane receptor tyrosine kinases [31,63,64] as well as with protein kinase C (PKC; [65]), a component downstream of the Fgf receptor signal transduction pathway [17]. Pdlim7 may be necessary as an adapter, bringing together transmembrane proteins and intracellular signal transducers. Without Pdlim7 function, the fin mesenchyme may not be competent to respond to either paracrine signals from the AER or autocrine signals, despite the expression of *fgf10 *and *fgfr1 *in the fin mesenchyme. The eventual loss of *fgfr2 *and misexpression of *pea3 *and *erm*, coupled with the lack of *fgf8 *expression, suggest a breakdown of Fgf response in the fin mesenchyme.

However, the Fgf pathway genes that remain active in the fin, such as *fgf10 *and *fgf24*, may be a consequence of Tbx5 misregulation caused by knock-down of Pdlim7. The ectopic activity of Tbx5 could account for the loss of specific mesenchymal and AER genes. Work by others has shown Tbx5 to function upstream of Fgf24, which may be a direct target, and Fgf10 during pectoral fin development (Fig. 8; [11,41]). *fgf10 *expression may be maintained in the fin mesenchyme by ectopic expression of *fgf24 *as a result of increased nuclear Tbx5 levels, despite the loss of AER signaling.

## Conclusion

Here we provide the first evidence for a role of a PDZ-LIM family member in vertebrate limb development. Pdlim7 is expressed in developing zebrafish pectoral fins and is required for normal outgrowth. In line with the model that Pdlim7 regulates Tbx5 transcriptional activity by altering its subcellular location, during fin development this regulation appears to have direct consequences on *fgf24*. Loss of Pdlim7 function results in ectopic *fgf24 *expression in the fin mesenchyme but lack of *fgf24 *induction in the AER, resulting in a breakdown of the Fgf signaling loop required for fin mesenchyme proliferation and outgrowth.

## Methods

### Zebrafish

Wild-type (TU) stocks were maintained at 28.5°C. Embryos were staged according to Kimmel et al. [66]. Embryos were cultured in 0.0045% phenylthiourea in Danieau buffer beginning at 24 hpf to inhibit pigmentation.

### Morpholino and mRNA injection

Antisense morpholino (MO) oligonucleotides were obtained from Gene Tools (Gene Tools, LLC, Philomath, OR). Pdlim7 MO2 was targeted to the splice donor site of exon 2 and detailed MO controls are described in Camarata et al. [38]. Embryos were injected at the one-cell stage and fixed at appropriate time points with 4% formaldehyde prepared from paraformaldehyde. Uninjected sibling embryos were fixed along with morpholino injected embryos as controls.

Zebrafish *pdlim7 *mRNA was synthesized using the mMessage mMachine kit (Ambion), as described [38]. Embryos were injected with 100 pg of *pdlim7 *mRNA at the one-cell stage and fixed at appropriate time points with 4% formaldehyde prepared from paraformaldehyde. Uninjected sibling embryos were fixed along with mRNA injected embryos as controls.

### In situ hybridization

Whole mount *in situ *hybridization was performed as previously described [38] using an Intavis Insitu Pro VSi (Koeln, Germany). Antisense RNA probes used were *pdlim7*, *tbx5 *[38], *hand2 *[42], *shh*, *msxc*, *msxd *[49], *fgf24 *[41], *pea3, erm *[67], *fgfr1*, *fgfr2 *[68], *versican *[69], and *fgf8*. *fgf10 *cDNA was cloned into Bluescript KS+ using primers previously described [18]. Embryos were imaged on a Leica MZ16 stereomicroscope fitted with a Leica DFC490 color camera using ImagePro MC (MediaCybernetics) software. Images were processed using Adobe Photoshop CS3.

### Embryo sectioning

Whole mount *in situ *hybridization using *pdlim7 *probe was performed on embryos fixed at 48 hpf. Embryos were allowed to sink in 30% sucrose, embedded in O.C.T. Compound (Tissue-Tek), and 10 micron sections obtained using a Leica CM3050 S cryostat. Sections were imaged on a Leica DMR upright microscope fitted with a QImaging Retiga-4000R Fast 1394 color camera using OpenLab software. Images were processed using Adobe Photoshop CS3.

### Immunohistochemistry and TUNEL

For detecting proliferating cells and developing somites simultaneously, 28-48 hpf fixed embryos were incubated in a block solution consisting of 10% sheep serum, 2 mg/mL BSA, and 0.2% saponin in PBS with Tween-20 (PBT) for 1 hour at room temperature. Next, the Anti-phospho-histone-h3 antibody (Anti-p-H3) (Millipore) was used at a 1:20 dilution, while the MF20 antibody was used at a 1:40 dilution in PBT with 0.2% saponin. Embryos were incubated in the primary antibody solution for 1 hour at room temperature or at 4 degrees Celcius overnight. Embryos were then washed in PBT multiple times before incubation for 1 hour at room temperature in secondary antibodies diluted at 1:100 in PBT with 0.2% saponin. For detection of apoptotic cells in whole mount embryos TUNEL staining was performed essentially as previously described [70]. Briefly, 28-48 hpf fixed embryos were incubated in proteinase K for 5 - 10 minutes, subjected to several washes of PBT, and subsequently incubated at 37°C in a TUNEL cell death detection reagent for 1 hour (in situ cell death Detection Kit-TMR Red, Roche Diagnostics). Embryos were then washed multiple times in PBT and then incubated for 1 hour at RT in the block solution prior to incubation with the MF20 antibody (developed by D. A. Fischman), which was obtained from the Developmental Studies Hybridoma Bank (University of Iowa), to visualize somites. Antibody block was made as previously described and MF20 was diluted to 1:40 in PBT with 0.2% saponin as described above. Confocal microscopy was performed on a Zeiss LSM510. Statistical significance between wild-type and MO2 injected embryos was determined using two-tailed student's t-test.

### Alcian blue staining

For cartilage analysis, zebrafish larvae were fixed at 96 hpf and treated with Alcian blue solution dissolved in 80% ethanol/20% glacial acetic acid (acid alcohol) for several hours or overnight. Larvae were destained in several washes of acid alcohol before being transferred to a 1% KOH:3% hydrogen peroxide solution for further clearing of pigment cells. Larval tissue was then digested in trypsin, followed by dissection of pectoral fin cartilages, which were then flat mounted and visualized on a Leica MZ16F.

## Authors' contributions

TC and HGS conceived and designed the experiments. TC, DS, TS, JK, and BH, performed the experiments. TC, DS, TS, JK, BH, and HGS wrote and edited the manuscript. All authors read and approved the final manuscript.

## Supplementary Material

Additional file 1 **Table S1**. Supporting quantitative data for p-H3 and TUNEL staining shown in Fig. 2. Click here for file
