# Supplementary material for: Pdlim7 is required for maintenance of the mesenchymal/epidermal Fgf signaling feedback loop during zebrafish pectoral fin development
Source: BMC Dev Biol. 2010 Oct 15;10:104. doi: 10.1186/1471-213X-10-104 (PMC2967529; doi:10.1186/1471-213X-10-104)
Supplement: Additional file 1 — Table S1. Supporting quantitative data for p-H3 and TUNEL staining shown in Fig. 2. [file 1471-213X-10-104-S1.DOC]

| p-H3 positive cells | | | | |
| --- | --- | --- | --- | --- |
| Stage | Class | Mean Value | n | p-value |
| 28 hpf | Wild-type | 9.6 | 5 | 0.077 |
|  | MO2 | 4.8 | 5 |  |
| 36 hpf | Wild-type | 13.25 | 4 | 0.036 |
|  | MO2 | 7 | 5 |  |
| 48 hpf | Wild-type | 21.8 | 5 | 0.001 |
|  | MO2 | 7.2 | 5 |  |
| TUNEL positive cells | | | | |
| Stage | Class | Mean Value | n | p-value |
| 28 hpf | Wild-type | 0.16 | 6 | 0.483 |
|  | MO2 | 0.5 | 4 |  |
| 36 hpf | Wild-type | 0 | 6 | 0.05 |
|  | MO2 | 1.4 | 5 |  |
| 48 hpf | Wild-type | 0.75 | 4 | 0.073 |
|  | MO2 | 2.85 | 7 |  |
